# Mal de Debarquement Syndrome: a survey on subtypes, misdiagnoses, onset and associated psychological features

**DOI:** 10.1007/s00415-017-8725-3

**Published:** 2018-01-05

**Authors:** V. Mucci, J. M. Canceri, R. Brown, M. Dai, S. Yakushin, S. Watson, A. Van Ombergen, V. Topsakal, P. H. Van de Heyning, F. L. Wuyts, C. J. Browne

**Affiliations:** 1 Antwerp University Research Centre for Equilibrium and Aerospace (AUREA) Antwerp, University of Antwerp, Antwerp, Belgium; 2 School of Science and Health, Western Sydney University, Sydney, NSW Australia; 3 School of Medicine, Western Sydney University, Sydney, NSW Australia; 4 Icahn School of Medicine, Mount Sinai Hospital, New York City, NY USA; 5 Prince of Wales Private Hospital, Sydney, NSW Australia; 6 University Department of Otorhinolaryngology and Head and Neck Surgery, Antwerp University Hospital, University of Antwerp, Antwerp, Belgium; 7 Translational Neuroscience Facility, School of Medical Sciences, UNSW Sydney, Sydney, NSW Australia

**Keywords:** Mal de Debarquement, Mal de Debarquement Syndrome, MdDS, Vestibular, Neuro-otology, Psychological component of MdDS

## Abstract

**Introduction:**

Mal de Debarquement Syndrome (MdDS) is a neurological condition typically characterized by a sensation of motion, that persists longer than a month following exposure to passive motion (e.g., cruise, flight, etc.). The most common form of MdDS is motion triggered (MT). However, recently it has been acknowledged that some patients develop typical MdDS symptoms without an apparent motion trigger. These cases are identified here as spontaneous or other onset (SO) MdDS. This study aimed to address similarities and differences between the MdDS subtypes. Diagnostic procedures were compared and extensive diagnostic guidelines were proposed. Second, potential triggers and associated psychological components of MdDS were revealed.

**Methods:**

This was a retrospective online survey study for MT and SO MdDS patients. Participants were required to respond to a set of comprehensive questions regarding epidemiological details, as well as the diagnostic procedures and onset triggers.

**Results:**

There were 370 patients who participated in the surveys. It is indicated that MdDS is often misdiagnosed; more so for the SO group. In addition to the apparent self-motion, both groups reported associated levels of stress, anxiety and depression.

**Discussion:**

It appears at present that both MdDS subtypes are still poorly recognised. This was the first attempt to evaluate the diagnostic differences between MdDS subtypes and to propose a set of comprehensive diagnostic guidelines for both MdDS subtypes. In addition, the current research addressed that associated symptoms such as stress, anxiety and depression should also be considered when treating patients. We hope this study will help the medical community to broaden their awareness and diagnostic knowledge of this condition.

**Electronic supplementary material:**

The online version of this article (10.1007/s00415-017-8725-3) contains supplementary material, which is available to authorized users.

## Introduction

Mal de Debarquement Syndrome (MdDS) is a neurological disorder, typically characterized by the perception of self-motion that persists for more than 1 month from an onset, which in most cases occurs after disembarkation from a vehicle (e.g., boat, cruise, train, plane). The phenomenon was first described by Erasmus Darwin in 1796 [1] and anchored by Brown and Baloh in 1987 as Mal de Debarquement [2–5]. Those cases with a symptomatic remission in 1 month from the onset are considered transient and hence named Mal de Debarquement (MdD) [3, 4]. The main features of MdDS are a constant sensation of rocking, swaying, bobbing and bouncing when walking, as well as continuing when lying down [3, 6]. These sensations are persistent while patients are awake and are independent on body position or movements. They are also accompanied by a myriad of symptoms such as heightened sensory sensitivity (e.g., photophobia), head pressure, nausea, agoraphobia, brain fog, associated migraine and fatigue, as well as depression and anxiety [5]. Interestingly, these symptoms often subside temporarily when patients are re-exposed to passive motion (e.g., driving or being a passenger in a car) [4, 5]. This temporary alleviation of symptoms is unique to MdDS patients and usually used for confirming a diagnosis of the condition [7].

In the past decade, there has been a growing interest in MdDS from the scientific community, as well as from patients. Despite some MdDS information that is available from the MdDS community, many patients still find it difficult to receive a clear diagnosis from a healthcare professional for timely treatment [8]. In general, MdDS is still considered a rare neurological disorder [5], and the prevalence of this condition has only been assessed in one study to date [9], where it was estimated to have an occurrence rate of 1.3% in a neuro-otological clinic. Currently, it is still unclear how many MdDS patients are misdiagnosed or undiagnosed. It is known that on average, MdDS patients undergo around 19 appointments with healthcare professionals before receiving a correct diagnosis [10], and that this diagnostic process can take as long as several years [11]. This is primarily due to unawareness and limited knowledge of MdDS amongst physicians or healthcare professionals, but also due to the lack of clear diagnostic guidelines [7]. There was only one publication by Van Ombergen et al. that set forth preliminary diagnostic guidelines based on a literature review [4]. Due to the lack of understanding of the condition when they developed the guidelines, specific aspects of MdDS such as onset types were not distinguished [2]. MdDS is often unremarkable in peripheral vestibular function [3, 4], and brain imaging studies. This makes the diagnosis even more challenging. According to recent publications, MdDS, regardless of the cause of onset, has been typically misdiagnosed as vestibular migraine [9], ‘atypical’ Ménière’s disease, general space and motion disorder, panic disorders and generalised anxiety disorders [8].

The past few decades has seen rapid growth in interest and knowledge regarding MdDS. As more patients have been evaluated, it has become clear that typical MdDS symptoms can occur from events other than motion or spontaneously. It is apparent that MdDS can be categorized into two subtypes according to the patient’s onset cause. From the literature, a less common and less acknowledged form of MdDS is non-motion or spontaneous/other (SO) onset MdDS. This type of MdDS can occur either in the complete absence of an event or trigger, uncharacterised by a specific motion-related event (spontaneous), or can be associated with stressful events such as surgery, trauma, childbirth and others. Although there might be a better term than spontaneous MdDS or SO MdDS to describe the non-motion triggered nature of MdDS, this term has been accepted differentially from MT MdDS. The literature about spontaneous MdDS is very limited and there is a need to develop clear diagnostic guidelines for this MdDS subtype. Aside from the differences in onset cause, MT and SO MdDS appear to be symptomatically identical [9] and respond similarly to at least one of the proposed treatments for MdDS [12]. However, a clear comparison between the two subtypes has yet to be done, in particular taking into account epidemiology, diagnostic procedures and potential triggers responsible for the onset.

It has been reported that MdDS patients have a high level of depression, poor quality of life and high illness intrusiveness [8], regardless of their onset type. Illness intrusiveness is considered an underlying determinant of the psychosocial impact of chronic diseases and illness, and it has been found to be correlated with poor quality of life [8]. Psychological and mood disorders (e.g., anxiety, depression) secondary to MdDS have been previously described in patients [6, 12]. It has been proposed that secondary mood disorders develop as a consequence of the continuous perception of motion, which may lead to mental strain [9, 13], as well as the frustration with the endless search for a correct diagnosis and the changes in lifestyle required to cope with the condition. MdDS is believed to be extremely debilitating, both physically and mentally [2, 3, 8–10]. The lack of awareness and recognition of MdDS from healthcare professionals further aggravates patient predisposition to the development of psychological disorders (e.g., anxiety and/or depression) [7]. Despite psychological symptoms being common, and considered part of the MdDS pathophysiology, it remains unclear if they are a pure consequence of MdDS inducement; or rather exist as vulnerability factors for developing the condition. Similarly, stress has also been known for negatively impacting MdDS patients [7]. It has long been known that stress has many physiological effects on the body [14] and it may be involved in influencing central vestibular and automatic functions in healthy individuals as well as in patients [15]. However, despite the potential role of stress in MdDS [7], the relevance of stress has never been closely evaluated. As a result, it remains unclear if it is a key trigger during MdDS onset, or if stress responses are triggered once the condition is established.

Observing the commonality of specific triggers across MdDS patients could help researchers understand why MdDS develops at a specific time. For example, it is possible that MT patients might have been exposed to similar passive motion before but yet they did not develop MdDS. As a result, the current study aimed to examine such factors as stress, depression and emotional status that may be equally important for eliciting MdDS. We also assess the prevalence of psychological comorbidities before and after MdDS onset. Based on previous studies [3, 9], it is reasonable to believe that psychological symptoms originate from MdDS. However, in this study, we also investigated if psychological disorders (e.g., being previously diagnosed with depression/being previously diagnosed with anxiety) were pre-existing conditions in MdDS patients. If true, this may help us to understand why there is a particular group of the population who seem more susceptible to MdDS.

To assess differences and similarities between MT and SO MdDS, specifically regarding diagnosis, onset and the major associated symptoms, two online surveys were made available to MT and SO MdDS patients. To date, some surveys have been popularized: the first by Gordon on transient MdD [16], followed by Hain et al. in 1999 [17] on MdDS. Epidemiological questionnaires and surveys on MdDS pathophysiology were also performed more recently [7, 10]. In this study, we aimed to provide a broad overview of MdDS and comprehensive diagnostic guidelines for both MdDS subtypes. The current study analysed diagnostic procedures, onset, and additional features associated with MdDS such as depression, anxiety and the role of stress.

## Methodology

### Ethical approval/study population and recruitment

Ethical approval was provided by the Ethics Committee of the University Hospital Antwerp Belgium (IRB number 15/44/454) and by the Western Sydney University Human Ethics Committee (H11962). Each respondent gave informed consent. All investigations have been conducted according to the principles expressed in the Declaration of Helsinki.

Patients diagnosed by specialists or believing to suffer from MdDS (also referred to as self-diagnosed patients) were recruited for the study. Patients were recruited across the USA, Europe, and Australia; however, respondents from Asia and South America were also able to access the study. MdDS patients were recruited through the Department of Otorhinolaryngology at the University Hospital of Antwerp, Belgium. Patients were also recruited globally through the main MdDS support groups: MdDS Australia Facebook Support Group, MdDS UK Facebook Support Group, website of MdDS Research Group at Mount Sinai Hospital, Western Sydney University MdDS Research Group Facebook page, website and Facebook of Vestibular Disorders Association (VEDA), and website and Facebook of Whirled Foundation and the REACT Community Facebook. A total of 370 respondents completed the surveys. Within these, 266 responded to the MT questionnaire and 104 to the SO questionnaire.

### Inclusion and exclusion criteria

Inclusion criteria: Patients reporting sensations of self-motion (rocking, swaying and bobbing) for longer than 1 month, where the symptoms could not be explained by another diagnosis. Only patients > 18 years old were included. Patients reporting MdDS symptoms after the exposure to passive motion, most frequently a boat trip, or travel over air or land were denoted as the motion-triggered group (MT group). Patients reporting similar symptoms without a clear motion event or any obvious cause were allocated to the spontaneous onset MdDS group. Patients reporting the initial symptoms after a strong emotional or stressful event (e.g., child birth, concussion, physical trauma, surgery, etc.) were defined as the other onset MdDS group. Both ‘Spontaneous’ and ‘Other’ onset MdDS patients were unified in one group, termed the SO group.

Self-diagnosed respondents were also included in the survey as numerous questions assessed their symptoms; we were able to screen them while also gaining information about their onsets.

Exclusion criteria: Patients < 18 years old. Patients reporting symptoms which do not fit with the MdDS guidelines [4].

### Questionnaires

The questionnaires were distributed online using Survey Monkey (MT group) and Qualtrics (SO group). The MT MdDS survey consisted of 51 questions and the SO MdDS survey consisted of 85 questions. More questions were made available to the SO group, as the respondents were re-directed to one of two specific categories: (1) spontaneous, and (2) other, according to their onset. Additionally, more extensive questions about hormonal profiles were distributed in the SO MdDS questionnaire. However, only the same questions were examined for both onset groups. The questions were divided into separate categories for both surveys: epidemiology (demographic details), diagnosis (i.e., who made the initial diagnosis, time frame before receiving the diagnosis, number of appointments), onset triggers (potential triggers related to the onset: events, hormonal fluctuations, medications, stress), symptom triggers (i.e., symptom fluctuation, assessments of potential triggers, susceptibility to visual inputs), hormonal influences and symptom management and treatment. This manuscript focuses on the diagnostic, psychological components of MdDS (stress; secondary mood disorders) and onset trigger data (questions available in Supplementary Material).

### Statistical analysis

Statistical analysis was performed with SPSS version 24 (IBM Corp). Chi Square was used for comparison between MT MdDS and SO MdDS groups.

## Results

### Epidemiology

The mean age was similar for both groups, i.e., 48.9 (SD 11.4) years for the MT group and 48.9 (SD 13.6) years for the SO group. A total of 370 surveys were collected, with 266 (71.9%) being the MT group, and 104 (28.1%) being the SO group. A female predominance was observed in both groups, with 242 female respondents (93.1%) in the MT group and 92 (88.5%) in the SO group. Half of all the respondents from both surveys were from North America (50.9% MT—51% SO), while 25% of the MT group and 24% of the SO were from Europe, 21.9 and 22.1% from Australia. A small number of respondents completed the surveys from Asia (0.8% MT—1% SO) and from South America (0.8% MT—1.9% SO). Respondents did not complete all the questions. As a result, the number of answers received per question and the respective percentage, is indicated for each question.

### Diagnosis

#### Initial diagnosis

Respondents from both groups were asked who initially diagnosed them with MdDS, and when comparing the two onset groups (MT versus SO), there was a significant difference between the two groups with respect to which health professional initially diagnosed their MdDS (*p* < 0.003). See Table 1.

**Table 1:** Initial diagnosis of MT and SO respondents expressed as the number of respondents (*n*) and percentage of respondents for both groups

Initial diagnosis	MT *n* (%)	SO *n* (%)
Self-diagnosed	125 (47)	33 (35.9)
ENT	61 (22.9)	19 (20.7)
Neurologist	42 (15.8)	25 (27.2)
Health care professionals (physiotherapists, chiropractors, physical therapists, nurses, etc.)	23 (8.6)	15 (16.3)
General physician/primary care physician	15 (5.6)	0 (0)
Total number of respondents that answered this question (%)	266 (100)	92 (88.5)

Self-diagnosis was the most common initial diagnosis in both groups, followed by ENT doctors, then neurologists for the MT group, and neurologists, then ENT doctors for the SO group

#### Diagnosis confirmation

After initial diagnosis, respondents were asked who confirmed their diagnosis. Only around one in five respondents [59 MT respondents (22.2%) and 17 SO respondents (18.5%)] received the MdDS diagnosis as their initial diagnosis. The remaining respondents subsequently consulted multiple healthcare professionals. ENT doctors and neurologists were among the majority of doctors who confirmed the diagnosis for the MT group [ENT = 69 (25.9%), neurologists = 68 (25.6%)]. For the SO group, a smaller number of ENT doctors (14–15.2%) confirmed the diagnosis, compared to neurologists (26–28.3%).

#### Medical appointments

In response to the following question: ‘provide an estimate of how many medical appointments you attended before your MdDS diagnosis’, the majority of the MT and SO group received their diagnosis from a healthcare provider within 2–5 appointments. However, the percentage of respondents between the two groups was statistically different (*p* < 0.024), especially for a single appointment (Table 2).

**Table 2:** Number of appointments attended in the search for a MdDS diagnosis expressed as the number of respondents (*n*) and percentage of respondents for both groups

Number of appointments	MT *n* (%)	SO *n* (%)
1	26 (17)	5 (6.7)
2–5	68 (44.4)	24 (32)
6–10	33 (21.6)	23 (30.7)
10–20	17 (11.1)	12 (16)
20–40	8 (5.2)	10 (13.3)
40 +	1 (0.7)	1 (1.3)
Total number of respondents that answered this question (%)	153 (57.1)	75 (72.1)

Respondents within the MT group had a higher chance of being diagnosed in fewer amount of appointments than those within the SO group

#### Number of appointments attended by self: diagnosed respondents

The number of self-diagnosed respondents was significantly different between the MT and SO subtype, with 112 (42.1%) respondents from the MT MdDS subtype and 40 (23.5%) respondents from the SO MdDS group (*p* = 0.002).

The majority of self-diagnosed MT respondents (59 responses—52.7%) attended 2–5 appointments, compared to a smaller number of the SO respondents (9 responses—22.5%). 10–20 appointments were reported among self-diagnosed MdDS respondents, with similar prevalence between MT group (11 responses—9.8%) and SO group (13 responses—32.5%).

#### Time frame before being diagnosed

The time before receiving a diagnosis after onset differed significantly between the MT and SO groups (*p* < 0.001) (Table 3) and in general, the results show that SO respondents wait a longer period of time to receive the correct diagnosis.

**Table 3:** Time before receiving MdDS diagnosis expressed as the number of respondents (*n*) and percentage of respondents for both groups

Time before receiving MdDS diagnosis	MT *n* (%)	SO *n* (%)
1–2 months	76 (34.1)	10 (12.8)
3–6 months	73 (32.7)	21 (26.9)
7–12 months	22 (9.9)	12 (15.4)
1–2 years	12 (5.4)	9 (11.5)
2 + years	17 (7.6)	15 (19.2)
5 + years	23 (10.3)	11 (14.1)
Total number of respondents that answered this question (%)	223 (83.8)	78 (75)

Respondents within the MT group had a higher chance of being diagnosed earlier than those within the SO group. With two-thirds of respondents within the MT group being diagnosed within 1–6 months from onset, and one-third of respondents within the SO group being diagnosed within 2–5 + years of onset

#### Other diagnoses

**Fig. 1:**
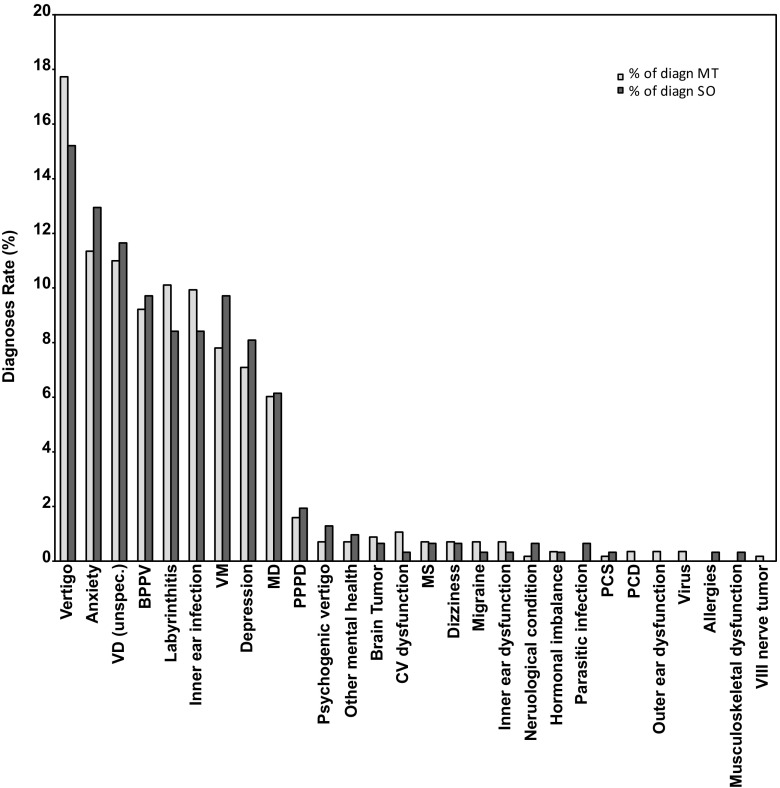
Various misdiagnoses received by respondents of the MT (light gray bars) and SO (dark gray bars) groups prior to MdDS diagnosis expressed as a rate (%) of received diagnoses. In both groups, vertigo was the most common misdiagnoses, followed by anxiety and then vestibular dysfunction. *VD* vestibular dysfunction (unspecified), *BPPV* benign paroxysmal positional vertigo, *VM* vestibular migraine, *MD* Ménière’s disease, *PPPD* persistent postural perceptual dizziness, *CV* cardiovascular, *MS* multiple sclerosis, *PCS* post-concussion syndrome, *PCD* posterior canal dehiscence, *VIII* vestibulocochlear

As presented in Fig. 1, various diagnoses were given to respondents within the MT and SO groups. The two groups received similar misdiagnoses. 263 (98.9%) responses were collected from the MT group and 104 (100%) from the SO group. All respondents received at least one misdiagnosis, with several reporting multiple misdiagnoses. On average, the respondents within the MT group received 2.1 different diagnoses and those within the SO group received three misdiagnoses. In summary, in both MT and SO, the term vertigo was used as a diagnosis and this was one of the most common misdiagnoses reported by participants, followed by anxiety and vestibular dysfunction (unspecified). After vertigo, anxiety and vestibular dysfunction, the next most common misdiagnoses for the MT group was labyrinthitis, inner ear infections, BPPV, vestibular migraine (VM) and depression. For the SO group, following are the most common misdiagnoses, BPPV, VM, labyrinthitis, inner ear infections and depression. No statistical differences were noted between the two groups.

#### Open-ended comments

Patients from both onsets subtypes were also given the opportunity to comment on their diagnostic experiences. 31 respondents within the SO group participated in this question, 71% of the comments described negative experiences, 16% described positive experiences and 13% of the responses were neutral regarding their quest for a diagnosis. 91 respondents within the MT also participated in this question, 58% of the comments described negative experiences, 20% described positive experiences and 22% of the responses were neutral.

### Onset

In Table 4, an overview of the respondents’ onset trigger (passive motion: car, bus, tram, flight, cruise), and of a potential trigger for the SO onset is represented as a percentage of respondents.

**Table 4:** Onset triggers reported by respondents within the MT and SO groups expressed as the number of respondents (*n*) and percentage of respondents for both groups

Triggers associated with MT onset	MT *n* (%)
Cruise	162 (60.9)
Flight	50 (18.8)
Combination of vehicles (e.g., flight and car; boat and car, etc.)	33 (12.4)
Train	6 (2.3)
Car	8 (3)
Bus	2 (0.8)
Simulator (virtual reality)	5 (1.9)
Total number of respondents	266 (100)

Possible triggers associated with SO onset	SO *n* (%)
Stress (psychological, physical)	10 (32.3)
Strong emotion	5 (16.1)
As a result of a previous vestibular disorder	3 (9.7)
Physical trauma (e.g., concussion)	7 (22.5)
Virus	2 (6.5)
Child birth/pregnancy + hormonal imbalances	3 (9.7)
Spontaneously (unable to recall a specific event)	1 (3.2)
Total number of respondents (%)	31 (29.80)

Cruising was the most common onset trigger for respondents within the MT group, followed by flights. Stress and physical trauma were the most common onset triggers for respondents within the SO group

### Additional features

#### Re-exposure to passive motion

Respondents were asked if there was an absence or a significant reduction of symptoms upon re-exposure to passive motion (e.g., driving or being a passenger in a car). 264 respondents from the MT group and 24 respondents of the SO group completed this question. Among those, 94.7% of the MT respondents and 91.7% of the SO respondents confirmed that they had a reduction of symptoms or full alleviation of symptoms when travelling in a moving car. Despite the small number of SO respondents, the majority of those who responded reported an alleviation of symptoms when exposed to passive motion, similar to the MT group. Combining the two groups together, the number of positive responses was significantly higher when compared to the respondents who did not report an improvement (*p* < 0.001).

#### Depression and anxiety

Respondents were asked if they had been diagnosed with depression and second, they were asked if they had been diagnosed with anxiety before or after developing MdDS in two distinct questions (Table 5). Respondents with depression were equally distributed and there was no significant difference between the two groups. However, the two groups differed significantly (*p* < 0.001) regarding the number of respondents diagnosed with anxiety before MdDS (20% MT—9% SO).

**Table 5:** Respondents’ diagnosis of depression and anxiety within the MT and SO groups expressed as the number of respondents (*n*) and percentage of respondents for both groups

	MT *n* (%)	SO *n* (%)
Depression		
Before MdDS	51 (19.3)	9 (28.1)
After MdDS	26 (9.8)	4 (1.4)
Never diagnosed with depression	187 (70.1)	19 (59.2)
Anxiety		
Before MdDS	53 (20.1)	3 (9.4)
After MdDS	35 (13.3)	12 (37.5)
Never diagnosed with anxiety	176 (66.7)	17 (53.1)
Total number of respondents	264 (99.2)	32 (30.7)

The majority of the MT and SO groups reported to have not been diagnosed with depression or anxiety

#### Psychological consequences of MdDS

Respondents who answered positively to depression and anxiety were asked whether they considered that their anxiety or depression were consequences of the syndrome per se. 107 (59.5%) of the MT respondents and 19 (70.4%) of the SO respondents replied that they believe MdDS was the cause of their psychological symptoms (e.g., depression, anxiety). No statistical difference was reported between the two groups. When considering the two groups together, a total of 291 answers were collected and 176 respondents answered that they believed MdDS was the cause of their psychological symptoms/conditions (*p* < 0.001).

#### Lifestyle changes

In response to the question regarding MdDS affecting respondents’ lifestyle, the following question was asked: ‘Do you feel that you have made lifestyle changes to avoid your triggers?’ (Symptom triggers such as exposure to bright, flickering light, excessive noise, going to the supermarket, etc.). This question was answered by 166 MT respondents (62.4%) and 36 SO respondents (34.6%). Both groups reported to have significant lifestyle changes to avoid triggers [166 (63.1%) MT respondents—36 (69.2%) SO respondents]. Considering the two groups together, a total of 215 respondents reported to have had to change their lifestyle (*p* < 0.001), 202 of which reported ‘a significantly changed lifestyle’. No statistical differences were found between the two groups (‘somewhat changed lifestyle’: 28.1% MT—26.9% SO; ‘little changes to lifestyle’: 1.5% MT—0% SO; ‘no changes to lifestyle’: 7.2% MT—3.8% SO).

Throughout both questionnaires, the respondents had the opportunity to add open-ended comments regarding anything they were willing to share about their MdDS experience. A great proportion of respondents expressed a high level of frustration and helplessness related not only to misdiagnoses and unawareness of MdDS in the medical community but also the debilitating nature of this condition and how much their lives had changed, “I regard myself as handicapped now”—SO respondent, “My whole life has significantly changed. I cannot go anywhere without my husband to hold on to. I am unable to travel on public transport on my own. I cannot go shopping on my own without a shopping trolley to hold on to. My whole life has changed.”—MT respondent. Patients also indicated that they were unable to work full time and live a normal life, others expressed their concerns about ageing with the condition, “My life barely resembles what it used to… I no longer travel, cannot see friends or have energy to do anything but work and come home to my family. I don’t go out, can’t physically exert myself, get seriously ill if I do exert myself, and can no longer do most of my hobbies or goals”—MT respondent, “It has changed my life. I am not able to do all the things I once enjoyed.”—SO respondent, “The biggest change to my lifestyle is a reluctance to go out unaccompanied due to the way I feel when I am walking. I feel much more secure with company and somebody to hold to alleviate the feeling of unsteadiness”—SO respondent.

#### Stress

**Fig. 2:**
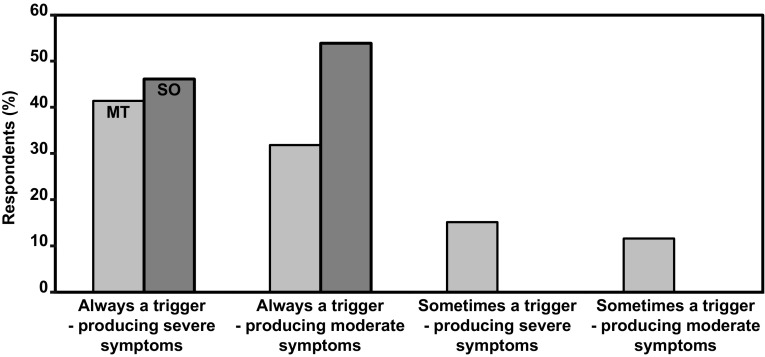
Stress as a trigger for increased symptoms (with various levels of aggravation) reported by respondents of the MT (light gray bars) and SO (dark gray bars) groups expressed as percentage of respondents who answered the question. In both groups, stress is viewed as a trigger that can produce a moderate to severe aggravation of symptoms

As shown in Fig. 2, stress is a significant symptom trigger for MT respondents (*p* < 0.001).

The SO group was asked if they were under stress during the potential onset. The question asked was: ‘Were you under stress during what you believe being the MdDS onset?’ The number of respondents answering positive was 21 (65%) (*p* < 0.001) and this question was answered by 31 SO respondents (20.2%).

## Discussion

In light of the difficulties involved in recruitment for a study concerning a rare condition, a multi-institutional collaboration was setup to collect data from MdDS respondents around the world. In total, 370 respondents completed either the MT or SO questionnaire. The current study is the largest in terms of MdDS respondents recruited to date, and is the only survey comparing MT versus SO subtypes. Furthermore, this study is the first to assess MdDS patients in a multicentred design and international setting. When considering the two onset subtypes, we have ascertained that the two categories clearly meet the clinical features of MT and SO MdDS. In line with published research [6], most MT respondents reported that their MdDS symptoms developed after a cruise, despite being on a cruise may be less frequent than being in a car, train or on a plane, time normally spent on a cruise is longer and during such type of travel, passengers are exposed to complex oscillation frequencies capable of disrupting the vestibular system and potentially the vestibular ocular reflex, following Dai’s theory [6]. Specifically, cruise ships normally rock from side to side at ≈ 0.2 Hz [12], which is known to induce motion sickness. We hypothesize that the exposure to such a strong stimulation and longer exposure times (typically), may be the reason why more people develop MdDS after disembarking a cruise ship. While 32% of the SO respondents developed MdDS after a period of psychological or physical stress or strong emotional experience, without the involvement of a motion event. It is possible that SO patients associate the onset of their disorder with a biographical event, and may appear ambiguous, nevertheless for this early stage of investigation, we believe it is important to collect as much information as possible about patients’ onset to identify any correlations between individuals within the subtype and to better objectify the differences between MT and SO subtypes.

We are aware that terms such as ‘psychological stress’ and ‘emotional experience’ are subjective terms with potentially broad interpretations. However, as these are the only events that these subsets of patients associate with their onset, they are of likely significance. A more extensive number of SO respondents is needed, as the SO survey was completed by a smaller number than the MT. A larger sample group could allow us to further define the psychological related aspects that SO patients attribute to MdDS onset.

Respondents from the MT and SO groups showed similar epidemiological results. The average age was 49 years old for both groups, with a strong female predominance. These results are comparable to a mean age of 45 years reported by other studies [3, 4, 7, 12, 17]. MdDS is a poorly understood disorder, and the lack of recognition and poor symptom management ultimately impact upon the patient’s mental state and lifestyle [9].

Patients learn to coexist with the syndrome. As reported by respondents from both groups, significant lifestyle changes were necessary, which in some cases had affected their employability, social life and ability to live, and what they consider a normal life. A large number of MT patients reported to have made significant adjustments to their lifestyle to avoid major triggers (such as exposure to bright, flickering light, excessive noise, going to the supermarket, etc.). The percentage of SO respondents implementing significant lifestyle changes was high (69.2%), similar to the MT group (63.1%). However, the number of total respondents in the SO group that responded to this question was much less than the MT group. Indeed, the open-ended comments received from both patients reveal the devastation that many MdDS experience after their onset. The majority of comments made by respondents in both groups referred to their high level of frustration and helplessness due to the great impairment that this condition has on their lives.

The lack of awareness among healthcare professionals contributes severely to misdiagnoses, and as a result some MdDS patients are unable to receive the correct diagnosis for long periods of time. In this survey, as previously stated, a high number of MT (47%) and SO (35.9%) respondents were self-diagnosed, meaning that they did not receive the MdDS diagnosis from any healthcare professional, despite their symptoms coinciding with the criteria for MdDS. Despite the potential risk of inclusion of non-MdDS sufferers, this category was maintained, based on previous research indicating that patients living with undiagnosed or highly debilitating conditions often resort to self-education through internet literature searches [9] and support group discussions [18]. As a result, the self-diagnosed MdDS patients in this study were patients who are still hoping to receive a confirmation of diagnosis but believed themselves to be suffering from MdDS. Results of this study found self-diagnosed SO respondents attended a higher number of medical appointments than the MT self-diagnosed group, indicating perhaps that the SO respondents have been continually seeking answers as they have not yet been officially diagnosed. On average, the SO respondents received a higher number of misdiagnoses, 3 different diagnoses, compared to 2.1 for the MT respondents. Similarly, both groups reported the same most prevalent misdiagnoses, which were vertigo and anxiety. Though these are not diagnostic terms, but rather symptoms, preliminary discussions with multiple patients revealed that many healthcare professionals often reported these terms as diagnoses, as a result we decided to include them in the questionnaire as diagnoses. The high number of patients misdiagnosed with vertigo and anxiety indicates that a great majority of healthcare professionals are not aware of vestibular disorders and more specifically MdDS, and are diagnosing patients with broad terms which focus on symptoms rather than an actual condition, and are using other diagnostic guidelines that do not include MdDS [19]. Following vertigo and anxiety, the most common misdiagnoses reported in the MT group was vestibular dysfunction, labyrinthitis, inner ear infection and BPPV. While the SO group reported diagnoses of vestibular dysfunction, VM, BPPV, labyrinthitis, inner ear infection, and lastly with depression. Related to the numerous misdiagnoses, more than 50% from both groups reported to have had negative experiences with health care providers. Most of the MT respondents were diagnosed by ENT doctors, followed by neurologists, whilst the opposite was true for SO respondents. This difference in diagnostic predominance between the ENT doctors and neurologists is probably due to the peculiarity of the SO group. The atypical onset is likely to have led to incorrect diagnoses. In addition, the MT respondents were found to have received the correct diagnosis earlier than the SO group. This data is understandable given the absence of clear diagnostic criteria for SO MdDS.

Thus, from the data obtained it is clear that diagnostic guidelines are needed to reduce misdiagnoses and improve patient’s management, and which also include the spontaneous or other forms of MdDS.

Hereto, we propose in Tables 6 and 7, new diagnostic guidelines for MT MdDS and SO MdDS, respectively. To be diagnosed with MT or SO MdDS, a patient should fulfil all the below mentioned criteria.

**Table 6:** New proposed MdDS diagnostic guidelines for patients with MT onset, adding new elements to Van Ombergen’s 2016 guidelines [4]

Chronic perception of motion (e.g., rocking dizziness, bobbing, swaying movements), that started after passive motion such as water, air and land travel, and that it is not affected by a patient’s position or movements
Symptoms lasting at least 1 month
Temporary relief of symptoms when re-exposed to motion (e.g., riding in a car), not necessarily the same motion that induced the onset, any passive motion
Normal inner ear function or non-related abnormalities as tested by electronystagmography (ENG)/videonystagmography (VNG) and audiogram should be present. However, if minor dysfunctions (e.g., minor hearing loss) are present, which do not implicate other vestibular pathologies, the patients can be included
Normal brain imaging study with standard MRI methods
Symptoms not better accounted for by other diagnoses made by a physician or health care professional

**Table 7:** New proposed MdDS diagnostic guidelines for patients with SO onset

Chronic perception of motion (e.g., rocking dizziness, bobbing, swaying movements), and that it is not affected by a patient’s position or movements
Symptoms lasting at least 1 month
Temporary relief of symptoms when re-exposed to motion (e.g., driving or being a passenger in a car)
Normal inner ear function or non-related abnormalities as tested by electronystagmography (ENG)/videonystagmography (VNG) and audiogram should be present. However, if minor dysfunctions (e.g., minor hearing loss) are present, which do not implicate other vestibular pathologies, the patients can be included
Normal brain imaging study with standard MRI methods
Symptoms not better accounted for by other diagnoses made by a physician or healthcare professional
Onset being spontaneous and not involving any exposure to passive motion

Symptomatic relief during passive motion was similarly reported for the MT and SO groups as presented in the results. This specific feature can clearly help distinguish MdDS patients from persistent postural perceptual dizziness (PPPD) (previously described as chronic subjective dizziness) [9, 20], visually induced dizziness (VID), phobic postural vertigo and motion sickness.

We are aware of the recent updates of the PPPD classification, which includes the previously named, chronic subjective dizziness, visual vertigo as well as phobic postural vertigo. Hereto, the most recent PPPD classification [20].

A. One or more symptoms of dizziness, unsteadiness, or non-spinning vertigo are present on most days for 3 months or more.
  1. Symptoms are persistent, but wax and wane.
  2. Symptoms tend to increase as the day progresses, but may not be active throughout the entire day.
  3. Momentary flares may occur spontaneously or with sudden movements.
B. Symptoms are present without specific provocation, but are exacerbated by:
  1. Upright posture,
  2. Active or passive motion without regard to direction or position, and
  3. Exposure to moving visual stimuli or complex visual patterns, although these three factors may not be equally provocative.
C. The disorder usually begins shortly after an event that causes acute vestibular symptoms or problems with balance, though less commonly, it develops slowly.
  1. Precipitating events include acute, episodic, or chronic vestibular syndromes, other neurologic or medical illnesses, and psychological distress.
  2. When triggered by an acute or episodic precipitant, symptoms typically settle into the pattern of criterion A as the precipitant resolves, but may occur intermittently at first, and then consolidate into a persistent course.
  3. When triggered by a chronic precipitant, symptoms may develop slowly and worsen gradually.
D. Symptoms cause significant distress or functional impairment.
E. Symptoms are not better attributed to another disease or disorder.

Despite the fact that MdDS shares similar features with these conditions now included and named as PPPD, (i.e., being triggered by visual stimuli, psychological stress and the development of anxiety and depression) [21], MdDS patients, regardless of their onset, are the only category of patients who experience an alleviation of symptoms when exposed to passive motion. Normally, PPPD patient’s symptoms are worse in motion [9], while VID patients are more prone to dizziness when observing their surroundings whilst moving (e.g., being in a car) due to busy or complex visual fields [22]. Finally, sufferers of phobic postural vertigo do not report any improvement of symptoms when exposed to passive motion. Yet, they do experience a reduction of symptoms once the mechanism of their complaints is explained to them and the patient feels reassured [23]. A potential theory regarding why MdDS patients report a reduction of symptoms when exposed to passive motion, may rely in the theory of adaptation to previous stimuli. As previously observed from functional magnetic resonance imaging (fMRI) and electroencephalography (EEG) studies [9], MdDS patients have been shown to have increased functional connectivity between multiple areas involved in spatial awareness (e.g., posterior sensory processing areas and the left entorhinal cortex (EC) [24], as well as within the amygdala). We hypothesize, as previously stated by Cha [24], that the exposure to passive motion generates vestibular and somatosensory signals, which produce frequencies of various amplitudes that are able to override or momentarily suspend any underlying oscillating rhythm perceived by the patients. It is apparent that more research is needed to understand the exact mechanism of this paradoxical relief of symptoms during re-exposure to motion. Novel neuroimaging research should be performed to address the phantom perception of motion experienced by all MdDS patients.

It was identified for most of the SO respondents that psychological stress, or physical or emotional trauma, was present at the time of their believed MdDS onset. The relation of the emotional event to the symptomatic onset is also observed in phobic postural vertigo patients [23]. To distinguish those two entities, we suggest using the criterion point number 3 (relief by passive motion).

## Stress and anxiety in MdDS

In general, more attention should be given to stress and its impact on MdDS development and symptomatology. The MT group reported to be significantly affected by moderate and severe stress, resulting in an aggravation of symptoms. In addition, the SO group is reported to be under severe stress during the period when the potential onset occurred. This strongly indicates that stress can be involved in aggravating symptoms and therefore be considered as a trigger, or it may be involved during onset in MT or SO group. Stress remains an extremely challenging factor in patients with vestibular disorders due to its physiological component and individuality aspect (e.g., age, gender, genetic factors, and early life experiences), as well as the variability of an individual’s perception over time [15]. Recently, the effects of stress on vestibular function and compensation have proven significant and are being increasingly recognised [15]. Several studies have observed stress in vestibular disorders such as chronic subjective dizziness (currently named PPPD) [24, 25], Ménière’s disease [26, 27] and vestibular migraine [26]. Other studies have reported that a chronic stress response was present in patients with a persistent vestibular dysfunction [27]. The vestibular system has been shown to have connections to the hypothalamic–pituitary–adrenal axis (HPA axis), the central stress response responsible for neuroendocrine adaptation to stressors [15, 28]. Balaban and Thayer in 2001 described the pathophysiologic mechanism of this problem [29]. The interaction between vestibular disorders and the psychiatric sphere is mediated through neurological pathways that are common to the control of the vestibular and autonomic systems, as well as emotional responses and anxiety [30].

**Fig. 3:**
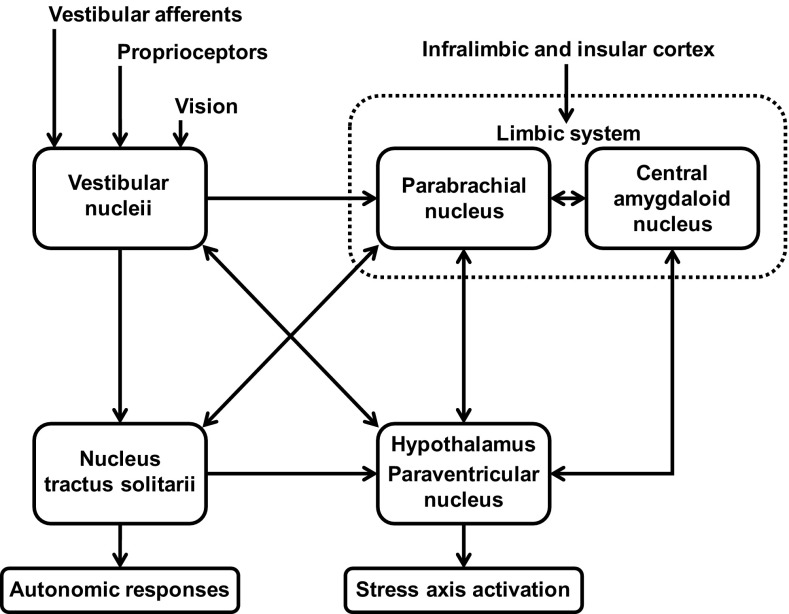
Schematic of the stress axis activation and its interrelation with the vestibular system. Adapted from [3]

As presented in Fig. 3, the connections from the vestibular nuclei to the parabrachial nucleus [31] are a direct link between the vestibular system and neural networks involved in expressing anxiety and emotion [32]. Stress may not only be affecting the patient’s symptoms, but also their physiological vestibular compensation mechanisms [14, 33]. In the study of Tschan and colleagues, stress, resilience and anxiety were considered among different vestibular patients as potential factors preventing vestibular compensation and rehabilitation [34]. Chronic stress is able to inhibit normal brain plasticity, leading to detrimental changes in the brain (e.g., hippocampus and prefrontal cortex) [33]. Potentially, chronic stress, as a result of MdDS, may be implicated in the pathophysiology of the disorder per se. Future studies should focus on assessing if MdDS patients report aberrant stress responses and consequently abnormal autonomic responses, ideally before and after a successful intervention. A disrupted HPA axis and autonomic response to stress could potentially be responsible for affecting an individual’s central compensation. Specifically, it could be responsible for the prolongation of MdD symptoms as well as having stress influencing symptoms and being involved in its onset.

## Additional features characterising MdDS

Anxiety and/or depression are recognised as the most common psychological symptoms associated with MdDS [4, 10]. MdDS patients have been previously described as being prone to developing psychological symptoms and disorders such as anxiety and/or depression due to the impact the condition has on a patient’s quality of life and lifestyle [10]. However, our study aimed not only to evaluate the prevalence of psychological disorders (specifically depression and anxiety) in MdDS patients, but also to evaluate whether predisposition factors were present before the syndrome onset. From our results, a smaller group of the SO respondents (3 respondents) reported to have been diagnosed with anxiety before MdDS compared to the MT group (53 respondents).

The development of anxiety after MdDS onset was relatively high in the SO respondents (37.5%). This reinforces our previous diagnostic argument, where the SO group’s levels of anxiety may be the consequences of a poorly understood and managed disorder as reported in previous research [3, 4]. However, given the limited number of responses, more data is required. For the MT group, on the other hand, anxiety for certain individuals can also be considered as a predisposing factor for developing MdDS. However, this should be further evaluated. Theoretically, generalised anxiety may contribute to develop an aberrant stress response [35], directly linked to changes in the sympathetic nervous system.

Depression was equally present in the MT and SO respondents prior to MdDS onset. Although the number of SO responses was limited, further studies with larger sample sizes are necessary to ascertain the relationship between depression and MdDS for this subtype.

Some anecdotal studies have previously asserted that depression was a predicting factor in vestibular disorders, but this was never proved [36]. However, if not considered as a predisposing factor, it is well-known that depression is associated in many patients affected by central vertigo/vestibular disorders (e.g., migrainous vertigo) [37]. A great number of MT respondents were diagnosed with depression after MdDS, confirming previous research [9]. Depression in MdDS patients may also be particularly important if considering its effect on cognitive functions [38]. Cognitive problems such as brain fog, and difficulties in focusing and concentrating, have been previously observed in MdDS [39], however, there has not been an established link between depression and the psychological component of MdDS with cognitive dysfunctions. We encourage future studies to examine closely the association between the two.

It is also very likely that the presence of psychological disorders may contribute to altering sensory information and interpretations in the brain regions (amygdala, insular cortex, cingulate cortex and prefrontal cortex) [40].

Previous studies focussing on brain functions in MdDS patients have reported hypometabolism in the left prefrontal cortex, left temporal cortex, right amygdala, and right insula [24]. The severity of MdDS bodily sensations and of vestibular dysfunction suggests that there may be a critical dependence of the brain and body upon vestibular input. It is known that there is a higher presence of depersonalization and derealisation symptoms in patients with vestibular dysfunctions, suggesting that the vestibular inputs are greatly contributing to the definition of self, in terms of the sense of where the body is in relation to the external environment [41]. The association of psychological disorders with MdDS may contribute to the neuroimaging differences previously observed.

Finally, our study suggests that MdDS is the cause for developing psychological disorders in both its subtypes, which is supported by the fact that antidepressants as well as sedative medications (e.g., benzodiazepines) are found to be helpful in MdDS patients and therefore often prescribed by healthcare professionals [39]. The use of such medication may also have an impact on stress levels, which from our study seems to be responsible for aggravating MdDS symptoms. A further and more detailed examination of the psychological component, psychological symptoms associated to MdDS and effect of stress on MdDS patients should be undertaken.

### Study limitations

Access to patients was limited to those active on social media and those who may have visited webpages that promoted our studies. The study was primarily limited by the inability of all respondents to recall or their lack of knowledge regarding specific details, particularly those connecting previous diagnoses and onsets. We are aware that the number of SO respondents was limited and less than the MT group. In addition to this, the definition between ‘other onsets’ and ‘spontaneous’ onsets could have been better clarified to the respondents of the SO survey, who for the first time had to self-define if they had ‘spontaneous’ or ‘other onset’ MdDS. Some respondents in this study were self-diagnosed; however, we assumed that many were able to diagnose themselves through resources available on the internet. Ideally, a larger patient pool where all respondents have received an official MdDS diagnosis would be preferable in future studies. Finally, regarding the psychological features of MdDS, we recognise that more detailed questions should have been asked. The current questionnaires only addressed the presence of two main psychological entities—anxiety and depression, before or after MdDS onset. For future studies, we encourage the distribution of validated questionnaires used for assessing both psychological features and psychosomatic symptoms in MdDS.

## Conclusion

This was the first online survey comparing two subtypes of MdDS. No major significant epidemiological differences were reported between MT and SO groups; both reported a female prevalence and the same mean age. MT patients are easier to diagnose than SO patients in terms of diagnostic procedures. Almost all the MT and SO respondents equally reported to have a reduction or absence of symptoms when re-exposed to passive motion. We encourage further EEG or fMRI studies to address this paradoxical perception of motion in MdDS patients. With this taken into account, we propose an updated version of the diagnostic criteria previously proposed by Van Ombergen and colleagues [4], to which we include symptom alleviation when exposed to motion, and also including the SO subtype.

Finally, these surveys allowed us to gain valuable information about other potential triggers and psychological features associated with MdDS. Stress was identified as a result of MdDS, a symptom aggravation factor, as well as a trigger for the onset especially in SO patients. Further studies should focus on measuring stress responses and autonomic reactions in MdDS. Depression and anxiety were identified as a clear consequence of MdDS and as a result, they should be taken into account for treatment options and patient management.

Overall, this study showed to be clinically relevant, providing more accurate diagnostic guidelines that can help establish an earlier and more accurate diagnosis in MdDS patients and provide more information about MT and SO MdDS subtypes.

## Electronic supplementary material

Below is the link to the electronic supplementary material.

Supplementary material 1 (DOCX 17 kb)
