# Supplementary material for: Mal de Debarquement Syndrome: a survey on subtypes, misdiagnoses, onset and associated psychological features
Source: J Neurol. 2018 Jan 5;265(3):486–99. doi: 10.1007/s00415-017-8725-3 (PMC5834551; doi:10.1007/s00415-017-8725-3)
Supplement: Supplementary file 1 — Supplementary material 1 (DOCX 17 kb) [file 415_2017_8725_MOESM1_ESM.docx]

**Supplementary Material**

Questions analysed and discussed in this manuscript

**Motion Triggered (MT) Questionnaire**

**1. BASIC INFORMATION
 1.2: Country/State/City:**

**1.3: Sex:**

**[ ] Male**

**[ ] Female**

**1.4: Date of Birth:**

**2. MDDS DIAGNOSIS**

**2.1: Who initially diagnosed you with MdDS:**

**[ ] Neurologist**

**[ ] Otolaryngologist**

**[ ] Physiotherapist**

**[ ] Self-diagnosed**

**[ ] Other [Free text box]**

**2.2: Who diagnosed you with MdDS after your initial diagnosis: (e.g. if you were self-diagnosed initially and received an official diagnosis subsequently) and was the specialist/health care professional confident in the diagnosis?**

**[ ] Neurologist**

**[ ] Otolaryngologist**

**[ ] Physiotherapist**

**[ ] Other [Free text box]**

**[ ] N/A as initial diagnosis was the only diagnosis**

**[ ] N/A as self-diagnosed is the only diagnosis**

**2.3: How long did it take to get the diagnosis of MdDS from the onset of your symptoms?**

**[ ] 1– 2 months**

**[ ] 3 – 6 months**

**[ ] 7–12 months**

**[ ] 1– 2 years**

**[ ] 2+ years**

**[ ] 5+ years**

**[ ] N/A Self diagnosed**

**2.4: If you have been diagnosed by a medical professional, provide an estimate of how many medical appointments you attended before your MdDS diagnosis (for example if you were sent to a physiotherapist, radiologist, etc.).**

**[ ] 1**

**[ ] 2-5**

**[ ] 6-10**

**[ ] 10-20**

**[ ] 20-40**

**[ ] 40+**

**2.5: If you are self-diagnosed, provide an estimate of how many medical appointments you have attended in the quest for an official diagnosis (for example if you were sent to a physiotherapist, radiologist, etc.).**

**[ ] 1**

**[ ] 2-5**

**[ ] 6-10**

**[ ] 10-20**

**[ ] 20-40**

**[ ] 40+**

**2.6: Prior to being diagnosed with MdDS, what other diagnoses have you received in response to your symptoms? Select all answers that apply:**

**[ ] Labyrinthitis**

**[ ] Inner ear infection**

**[ ] Depression**

**[ ] Anxiety**

**[ ] Posterior canal dehiscence**

**[ ] Brain tumour**

**[ ] Meniere’s disease**

**[ ] Vestibular dysfunction**

**[ ] Vestibular migraine**

**[ ] Vertigo**

**[ ] Psychogenic vertigo**

**[ ] Persistent postural-perceptual dizziness (PPPD)**

**[ ] Benign paroxysmal positional vertigo (BPPV)**

**[ ] Other [Free text box]**

**2.7: Is there anything you would like to add about your MdDS diagnosis or any experience that you feel is appropriate to this section? [Free text box]**

**3. MDDS ONSET AND SYMPTOMS**

**3.1: To the best of your knowledge, what was the motion event that induced your MdDS? Select one answer:**

**[ ] Short cruise (less than a day)**

**[ ] Long cruise (more than a day)**

**[ ] Short airplane flight (<3 hours)**

**[ ] Long airplane flight (>3 hours)**

**[ ] Short Train ride (<3 hours)**

**[ ] Long Train ride (>3 hours)**

**[ ] Short Car ride (<3 hours)**

**[ ] Long Car ride (>3 hours)**

**[ ] Short Bus ride (<3 hours)**

**[ ] Long Bus ride (>3 hours)**

**[ ] Short Tram ride (<3 hours)**

**[ ] Long Tram ride (>3 hours)**

**[ ] Fairground/theme park ride**

**[ ] Other [Free text box]**

**3.14: Are you prone to getting anxious or having depressive episodes?**

**[ ] Before and after MdDS onset**

**[ ] Before MdDS only**

**[ ] After MdDS onset only**

**[ ] No**

**3.15: Have you been diagnosed with depression?**

**[ ] Yes, before MdDS onset**

**[ ] Yes, after MdDS onset**

**[ ] No**

**3.16: Have you been diagnosed with an anxiety disorder?**

**[ ] Yes, before MdDS onset**

**[ ] Yes, after MdDS onset**

**[ ] No**

**3.17: Do you consider that your depressive or anxiety symptoms are a consequence of your MdDS?**

**[ ] Yes**

**[ ] No**

**4. SYMPTOM TRIGGERS**

**4.1: What are the triggering factors that would make your symptoms worse?**

**For each please select either (not a trigger / sometimes a trigger (producing moderate symptoms) / sometimes a trigger (producing severe symptoms) / always a trigger (producing moderate symptoms) / always a trigger (producing severe symptoms) for all triggers below: *the manuscript only discusses the STRESS responses**

**4.2: Do you feel that you have made lifestyle changes to avoid your triggers?**

**[ ] Yes, I feel like my lifestyle has significantly changed because of this condition**

**[ ] Yes, I feel like my lifestyle has somewhat changed because of this condition**

**[ ] No, I continue to live my life as per usual**

**[ ] No, this condition has little effect on my lifestyle**

**Spontaneous/Other Onset (SO) Questionnaire**

**1. BASIC INFORMATION**

**1.2: Country/State/City:**

**1.3: Sex:**

**[ ] Male**

**[ ] Female**

**1.4: Date of Birth:**

**2. MDDS DIAGNOSIS**

**2.1: Who initially diagnosed you with MdDS:**

**[ ] Neurologist**

**[ ] Otolaryngologist**

**[ ] Physiotherapist**

**[ ] Self-diagnosed**

**[ ] Other [Free text box]**

**2.2: Who diagnosed you with MdDS after your initial diagnosis: (e.g. if you were self-diagnosed initially and received an official diagnosis subsequently) and was the specialist/health care professional confident in the diagnosis?**

**[ ] Neurologist**

**[ ] Otolaryngologist**

**[ ] Physiotherapist**

**[ ] Other [Free text box]**

**[ ] N/A as initial diagnosis was the only diagnosis**

**[ ] N/A as self-diagnosed is the only diagnosis**

**2.3: How long did it take to get the diagnosis of MdDS from the onset of your symptoms?**

**[ ] 1– 2 months**

**[ ] 3 – 6 months**

**[ ] 7–12 months**

**[ ] 1– 2 years**

**[ ] 2+ years**

**[ ] 5+ years**

**[ ] N/A Self diagnosed**

**2.4: If you have been diagnosed by a medical professional, provide an estimate of how many medical appointments you attended before MdDS was diagnosed (for example if you were sent to a physiotherapist, radiologist, etc.).**

**[ ] 1**

**[ ] 2-5**

**[ ] 6-10**

**[ ] 10-20**

**[ ] 20-40**

**[ ] 40+**

**2.5: If you are self-diagnosed, provide an estimate of how many medical appointments you have attended in the quest for an official diagnosis (for example if you were sent to a physiotherapist, radiologist, etc.).**

**[ ] 1**

**[ ] 2-5**

**[ ] 6-10**

**[ ] 10-20**

**[ ] 20-40**

**[ ] 40+**

**2.6: Prior to being diagnosed with MdDS, what other diagnoses have you received in response to your symptoms? Select all answers that apply:**

**[ ] Labyrinthitis**

**[ ] Inner ear infection**

**[ ] Depression**

**[ ] Anxiety**

**[ ] Superior / Posterior canal dehiscence**

**[ ] Brain tumour**

**[ ] Meniere’s disease**

**[ ] Vestibular dysfunction**

**[ ] Vestibular migraine**

**[ ] Vertigo**

**[ ] Psychogenic vertigo**

**[ ] Persistent postural-perceptual dizziness (PPPD)**

**[ ] Benign paroxysmal positional vertigo (BPPV)**

**[ ] Other [Free text box]**

**2.7: Is there anything you would like to add about your MdDS diagnosis or any experience that you feel is appropriate to this section? [Free text box]**

**3. MDDS ONSET AND SYMPTOMS**

**3.1: Do you think your MdDS was triggered by an event, which was not motion, for example: trauma, concussion, childbirth, strong emotion (other onset); or did the onset of your MdDS seem to have no obvious cause (spontaneous onset)? Your answer will direct to you specific ‘other’ or ‘spontaneous’ onset questions.**

**[ ] I think that my MdDS onset was triggered by an event which is not considered passive motion. (‘other’)**

**[ ] I think that I had a spontaneous MdDS onset, as I cannot recall a specific event. (spontaneous)**

**FOR SUBJECTS ANSWERING ‘OTHER’:**

**OTHER event 3.2: To the best of your knowledge, what was the event that induced your MdDS?**

**[ ] Concussion**

**[ ] Trauma (physical or psychological)**

**[ ] Childbirth**

**[ ] Pregnancy**

**[ ] Strong Emotion**

**[ ] Other [Free text box]**

**OTHER event 3.5: Were you under a lot of stress when symptoms first appeared?**

**[ ]Yes**

**[ ] No**

**[ ] Not sure**

**OTHER event 3.6: Were you depressed when symptoms first appeared?**

**[ ]Yes**

**[ ] No**

**[ ] Not sure**

**OTHER event 3.23: Are you prone to getting anxious or having depressive episodes?**

**[ ] Before and after MdDS onset**

**[ ] Before MdDS only**

**[ ] After MdDS onset only**

**[ ] No**

**OTHER event 3.24: Have you been diagnosed with depression?**

**[ ] Yes, before MdDS onset**

**[ ] Yes, after MdDS onset**

**[ ] No**

**OTHER event 3.25: Have you been diagnosed with an anxiety disorder?**

**[ ] Yes, before MdDS onset**

**[ ] Yes, after MdDS onset**

**[ ] No**

**OTHER event 3.26: Do you consider that your depressive or anxiety symptoms are a consequence of your MdDS?**

**[ ] Yes**

**[ ] No**

**FOR SUBJECTS ANSWERING ‘SPONTANEOUS’:**

**SPONTANEOUS 3.4: Were you under a lot of stress when symptoms first appeared?**

**[ ]Yes**

**[ ] No**

**[ ] Not sure**

**SPONTANEOUS 3.5: Were you depressed when symptoms first appeared?**

**[ ]Yes**

**[ ] No**

**[ ] Not sure**

**SPONTANEOUS 3.17: Do you feel better or normal when you are riding in a car?**

**[ ] Yes**

**[ ] No**

**SPONTANEOUS 3.21: Are you prone to getting anxious or having depressive episodes?**

**[ ] Before and after MdDS onset**

**[ ] Before MdDS only**

**[ ] After MdDS onset only**

**[ ] No**

**SPONTANEOUS 3.22: Have you been diagnosed with depression?**

**[ ] Yes, before MdDS onset**

**[ ] Yes, after MdDS onset**

**[ ] No**

**SPONTANEOUS 3.23: Have you been diagnosed with an anxiety disorder?**

**[ ] Yes, before MdDS onset**

**[ ] Yes, after MdDS onset**

**[ ] No**

**SPONTANEOUS 3.24: Do you consider that your depressive or anxiety symptoms are a consequence of your MdDS?**

**[ ] Yes**

**[ ] No**

**4. SYMPTOM TRIGGERS**

**BOTH SPONTANEOUS AND OTHER:**

**4.1: What are the triggering factors that would make your symptoms worse?**

**For each please select either (not a trigger / sometimes a trigger (producing moderate symptoms) / sometimes a trigger (producing severe symptoms) / always a trigger (producing moderate symptoms) / always a trigger (producing severe symptoms) for all triggers below: *the manuscript only discusses the STRESS responses**

**4.2: Do you feel that you have made lifestyle changes to avoid your triggers?**

**[ ] Yes, I feel like my lifestyle has significantly changed because of this condition**

**[ ] Yes, I feel like my lifestyle has somewhat changed because of this condition**

**[ ] No, I continue to live my life as per usual**

**[ ] No, this condition has little effect on my lifestyle**
